# Multimedia Knowledge Translation Tools for Parents About Childhood Heart Failure: Environmental Scan

**DOI:** 10.2196/34166

**Published:** 2022-03-21

**Authors:** Chentel Cunningham, Hyelin Sung, James Benoit, Jennifer Conway, Shannon D Scott

**Affiliations:** 1 Faculty of Nursing University of Alberta Edmonton, AB Canada; 2 Department of Pediatrics Faculty of Medicine and Dentistry University of Alberta Edmonton, AB Canada

**Keywords:** environmental scan, pediatrics heart failure, parent audience, knowledge translation, web-based educational tools

## Abstract

**Background:**

Childhood heart failure is a factor in many hospital admissions each year. It can impose a steep learning curve for parents who need to learn the key information to care for their child at home. In this study, we conducted an environmental scan to identify and assess web-based knowledge translation tools about childhood heart failure for parent audiences developed within North America.

**Objective:**

The aim of this study is to inventory tools publicly available to parents about childhood heart failure from popular web-based venues, assess how each tool communicates health information, and explore how they were developed.

**Methods:**

Our search strategy included two commonly used multimedia-based platforms: two app stores (Google Play and Apple App Store) and one search engine (Advanced Google Search). Common search terms were used, and results were uploaded to Microsoft Excel for screening between 2 reviewers. The inclusion criteria for the tools were as follows: content focused on educating parents about their child’s heart failure, developed in the English language, and originating within Canada and the United States. A total of 2 reviewers screened the app store and internet search results for relevant tools. Each tool was assessed using the Suitability Assessment of Materials (SAM), a validated tool that objectively assesses the suitability of how health information is communicated to a particular audience. Key informants who were involved in tool development were identified and invited for a qualitative interview using a semistructured format to provide data about the development process. Key themes were identified in the semistructured interview process.

**Results:**

Frequencies and SAM percent ratings of eligible tools were reported. No apps exist for parents relating to pediatric heart failure. Overall, 17 relevant internet tools were identified, and their suitability was assessed for the parent audience. Most tools scored well in layout and type, but they scored lower in readability and graphics. Qualitative interviews with key informants revealed three key themes: timely and introductory knowledge, credible and trustworthy knowledge, and challenges and evolution in knowledge.

**Conclusions:**

This is the first environmental scan looking for parent tools relating to childhood heart failure in Canada and the United States. Findings from this study reveal that there are no apps on this topic and there is a small number of tools for parents on the internet (n=17). Using the SAM, no tools scored in the superior range, and further work in knowledge translation strategies needs to be done in this area to improve more effective education to parents and caregivers who have a child with heart failure. These findings will inform the development of a new resource on children’s heart failure that targets parents and caregiver audiences.

## Introduction

### Background

Parents who have a child with heart failure need understandable and reliable knowledge. Approximately 11,000 to 14,000 annual pediatric hospitalizations in the United States are due to children’s heart failure, with 87% of all initial cases diagnosed after an exacerbation in heart failure symptoms requiring invasive, life-saving medical intervention [1]. Heart failure in children can invoke uncertainty, heighten stress levels, and impose a steep learning curve on parents.

Since the release of North American evidence-based guidelines [2,3], more children with heart failure have been surviving, and parents have been caring for them in the outpatient setting. Parents are in the unique position of being termed *proxy health information seekers*, as they require advanced and ongoing information to provide day-to-day management for their ill child [4,5]. Aside from their health care team, a source where parents rely heavily upon for health information to make daily decisions about their child’s care is the internet [5]. Parents who have children with chronic health conditions have identified they require adequate and appropriate information to care for their child [6]. However, despite the call for this necessary information among parents, the literature still suggests that they feel generally unsupported in their quest for health information [7]. Undoubtedly, this need exists for parents of children with heart failure given the scarce amount of literature on this topic.

Multimedia-based educational tools (eg, e-books, apps, videos, and whiteboard animations) posted on the internet are novel strategies that can fill this knowledge gap by providing easy to access educational content to parents and caregivers who need it [8]. These tools have the ability to creatively accentuate evidence-based health information, resulting in better uptake by parent audiences [9]. They positively influence learning styles by providing complex information that is palatable, relevant, and understandable [10]. Knowledge translation tools have been created for parent audiences in other contexts and have been shown to provide understandable, accessible, and evidence-based knowledge that helps improve care [11]. Multimedia-based knowledge translation tools have not been widely explored in the context of childhood heart failure. To date, there is currently no understanding of what web-based knowledge translation tools exist for parent audiences about pediatric heart failure and how they are rated in terms of how they communicate medical information to parent audiences.

### Objectives

Our research seeks to better understand what tools are currently available for parents who have a child with heart failure and to assess each tool’s ability to enhance their knowledge. Therefore, the aim of our study is to understand what publicly available educational tools are available to parents of children with heart failure on the web and app stores.

## Methods

### Overview

The environmental scan (ES) methodology is used to scan the *scan the environment* in an organized manner for gray information pertaining to a specific topic or context [12]. Our ES was conducted in 4 stages searching Canadian and US-based educational tools for children’s heart failure that targeted parental audiences in June 2020.

A multimedia tool was included if it (1) was developed in either Canada or the United States, (2) focused solely on children’s heart failure content, (3) was developed in English language, and (4) targeted a parent or caregiver audience. Tools were only included if they were from Canada and the United States to preserve the feasibility of the study. Duplicate tools were excluded. Given the anticipated limitation in tools, a date range was not applied to the internet search to maximize our findings.

Data collection occurred in four separate phases: (1) app search, (2) internet search, (3) Suitability Assessment of Materials (SAM) evaluation, and (4) key informant interviews. Key informant interviewers serve to augment the findings of the search as they will provide richer detail about each resource’s development process. Multimedia Appendix 1 outlines the screening process of the app and internet search.

### Ethics Approval

As our study included a qualitative interview component with key informants, ethical approval was obtained from the University of Alberta Research Ethics Office (Pro00106559).

### Phase 1: App Search

Two app stores (Apple App Store and Google Play) were searched using the broad layman search term *heart failure* by the primary researcher (CC). Using the same term, a second researcher (JB) used a web scraping search strategy to ensure comprehensiveness. Web scraping is the systematic process of using a web *bot* (or software agent) to produce more comprehensive search results [13]. Searches were limited in the advanced search function to either Canada or the United States, totaling 2 searches. Modeled after previous ES methods [14], only the first 50 apps from the Canadian and US search in each app store were archived for review. The primary reviewer (CC) complied all the internet and app results into Microsoft Excel spreadsheets for screening. Screening for eligibility was completed by the primary reviewer, and all data were verified independently by a second reviewer (HS) to ensure accuracy.

### Phase 2: Internet Search

The primary reviewer (CC) performed the search using three broad laymen’s search terms in the *all these words* function of Google Advanced Search: (1) *child heart failure*, (2) *pediatric heart failure*, and (3) *child heart failure guidelines*. The primary reviewer ran each term separately for each country (eg, Canada or the United States), resulting in 6 separate searches. Other strategies to increase search results included disabling cookies and turning off personalization to help reduce search bias. Again, no date restriction was placed to maximize our search results. To keep the search feasible, the search was limited to English language tools and within Canada and the United States. Another reason to limit the search to any North American tools was to tailor our findings to apply to an educational tool we are developing about children’s heart failure that will be used in this area.

Similar to other ES methods [12], the first 100 webpage results from each search string were archived using screenshots and uploaded into a Microsoft Excel spreadsheet for screening by two reviewers (CC and HS). Adapted from methods used for data extraction and screening in systematic review processes to avoid data extraction errors [15], CC completed the initial screen and data extraction, and HS verified all data line by line. All discrepancies in data extraction and relevancy were flagged and discussed between the 2 reviewers, with no disagreements needing to be brought forth to the senior author (SDS). To increase the quality and accuracy in data collection, CC (an experienced pediatric heart failure clinician) educated the second reviewer about children’s heart failure [15]. All included internet educational tools were downloaded and examined in detail.

To prevent missing any relevant educational tools in the search process, the primary reviewer also consulted with a subject expert (JC) in the field of pediatric heart failure (eg, pediatric cardiologist) to review the list of screened websites, identifying any further relevant tools that may exist but were missed. An additional tool was identified by our subject expert (April 2020). This tool was posted to the web after the date of our initial search, and it was added to our relevant list of tools for health literacy appraisal.

Descriptive statistics and frequencies in Microsoft Excel were used to analyze the characteristics of both app and internet resources. A list of any relevant web-based tools in either the app or internet search was reported for the apps and web-based tools.

### Phase 3: SAM

All relevant internet and app resources that met the inclusion criteria were downloaded in full and scored independently by two reviewers (CC and HS) using the SAM scoresheet. The SAM assessment is a validated tool, developed by experts in health education for adults, that assesses the readability, usability, and suitability of health information [16]. SAM evaluation can pinpoint specific strengths or deficiencies in educational materials or compare different education materials for specific patient populations and suggest areas of improvement or refinement [16]. This method of scoring has not been applied in the pediatric heart failure context but in other pediatric contexts [17,18]. The original SAM scoring tool was developed by Doak et al [16] and was subsequently adapted with permission by Smith [19]. The version developed by Doak et al [16] includes 6 domains with 22 subfactors. The updated version by Smith [19] includes the 6 domains but with only 21 subfactors, omitting the scope evaluation within the content section. The modified version by Smith [19] was used, as the scope of this assessment was already tailored to include only tools about children’s heart failure that specifically educated the parental audience.

A rating score was obtained from each assessment (not suitable=0, adequate=1, and superior=2). Scores were then transformed into percentages (percent ratings; eg, 0%-39%=not suitable material, 40%-69%=adequate material, and 70%-100%=superior material). A rating of not suitable would indicate that a web-based resource requires some refinement to make it more suitable for the intended audience, whereas a superior rating would indicate that no further refinements are needed and a high level of health literacy [16]. Resources were not excluded from inclusion based on their SAM score, but rather the assessment was completed to provide a sense of the overall scope and quality of educational content that is available to parents who care for a child with heart failure. To ensure minimal bias in the review process [20], CC and HS both independently SAM rated each relevant educational tool, and then scores were disclosed and discussed. Any highly discrepant scores (>10 points difference) were discussed in detail among reviewers to understand the large variability in scores (eg, errors in scoring). Given the subjective nature of this scoring tool, an average overall rating between both reviewers for each domain was generated, giving an average SAM score for each resource.

### Phase 4: Key Informant Qualitative Interviews

Key informant interviews were conducted to complement the SAM ratings and add depth about the characteristics, distribution, and development process of each tool. Interviews with key interviewees were conducted by CC who contacted organizations from the information provided on the webpage. To maximize the number of informant responses, 3 attempts were made to contact each key informant (n=17) either by phone or by the email provided on the tool’s webpage. This approach was modeled after the method developed by Dillman [21] for achieving responsiveness in the context of surveys. All interviews were conducted and recorded using the Zoom video conference platform [22]. All interviews were live coded to allow for context and meaning to be present in the results [23]. All participants provided written consent before the interview.

Data collection and analysis occurred iteratively, allowing for a more precise and purposeful process. The number of interviews achieved was not decided based on data saturation but on the positive responses accepting the invitation to participate in a qualitative interview by key informants who played an integral role in tool development.

Thematic analysis was used to synthesize and identify common themes among key informants described in the semistructured interviews. Thematic analysis was modeled after the study by Braun and Clarke [24]. Outlined in their approach are four key stages: familiarization with the data, initial coding, searching for categories among the initial open codes, and constructing final major themes that best represented the data. A data-driven inductive approach was used to link the developed codes and themes to the data themselves [25]. The interviewer became immersed in the data through repeated listening of the recorded video interviews with live coding into summary tables. Codes remained genuine as they stayed as close to the participants’ own words. Codes started more general and became more focused as they were grouped into categories and then major themes. All codes and videos were then re-examined to ensure consistency and accuracy of the interpretation.

## Results

### Overview

A detailed flowchart outlining the screening process is presented in Multimedia Appendix 1. The screening and SAM ratings occurred over a 9-month period (July 2020 to March 2021).

### Phase 1: App Search

The app search was conducted in July 2020. In total, 112 apps were retrieved, 89 from the layman search strategy, and 24 additional from the scraper method. Unfortunately, no apps met the inclusion criteria, highlighting a knowledge gap in this platform for parents and caregivers about children’s heart failure.

### Phase 2: Internet Search

The internet search was completed in August 2020. A combined total of 575 websites were retrieved across 6 search terms. Screening of the 455 websites occurred between two screeners (CC and HS). Details of the included web-based pediatric heart failure tools are shown in Multimedia Appendix 2. A total of 16 relevant tools met the inclusion criteria, 13 from the United States and 3 from Canada. An additional relevant tool from the United States was identified following consultation with a subject expert (JC) in the field of children’s heart failure. This tool was not missed in the original search; it was developed and posted on the internet after August 2020. The most relevant internet tools were in the form of webpages (n=13) and handouts (n=3). The content for the relevant tools focused on a varying range of information (eg, general information, symptoms, treatment strategies, and testing).

### Phase 3: SAM Evaluation Rating Scores

The average overall SAM factor rating between the 2 reviewers ranged from a low suitability score of 38% (16/42) to a high score of 62% (26/42; Multimedia Appendix 3). The total possible SAM suitability scores were out of 42 (100%). No tool scored 100% (26/26). Overall, 15 tools’ ratings were in the adequate range (40%-69%), and 2 tools’ ratings were in the not suitable range (0%-39%). No tools scored within the superior range (70%-100%).

Each tool was scored individually according to each of the 6 SAM factors in each domain (eg, content, literacy demand, graphics, layout and typography, learning stimulation and motivation, and cultural appropriateness). Raw scores for each factor of the 17 tools were combined on each SAM factor, and a percentage score was calculated, demonstrating the overall current state of web-based tools included in this ES (Multimedia Appendix 4). Overall, most of the tools had a higher reading level than recommended, averaging over a ninth-grade reading level (13/17, 76%). Layout and type scores were all within the superior range—typography (17/17, 100%), layout (10.5/17, 62%), and subheadings (7/17, 41%). In contrast, all graphic scores were in the not suitable range—cover graphics (12.5/17, 74%), type of illustrations (10.5/17, 62%), relevance of graphics (10.5/17, 62%), graphic explanation (17/17, 100%), and graphic caption (16/17, 94%).

### Phase 4: Key Informant Interviews

#### Overview

Key informant interviews were conducted between April and June 2021. Of the 17 relevant webpage educational tools, only 16 (94%) had contact information available. In addition, 1 tool only included a *customer support* tab as opposed to a contact tab (eg, Contact Us or phone number). When the customer support tab was clicked on, the researcher was directed to a generic table of contents related to the website with no further contact information provided. After 3 attempts, 41% (7/17) of the organizations did not respond. In addition, 29% (5/17) of the organizations declined an interview with the rationale that their tool was developed by an outside vendor (n=4) or that the individual who made the tool was no longer employed at the organizations (n=1). Of the key informants who agreed to an interview, an average of 2 attempts were made before a response was received. Of the 17 key organizations, 4 (24%) agreed to participate in a qualitative interview. Moreover, 3 interviews had 1 participant, and the fourth interview had 3 participants. From all 4 interviews, interviewees were either medical professionals (n=4) or employed in leadership roles within the organization (n=2; eg, manager or director).

In total, 3 major themes arose from the semistructured qualitative interviews, which focused on the content, knowledge distribution and development process, and perceived impact. These three major themes are as follows: timely and introductory knowledge, credible and trustworthy knowledge, and challenges or evolution. Interviews were assigned a reference marker (eg, I2) for quotes present in support of the themes identified in our results.

#### Timely Introductory Knowledge

Participants in this study agreed that the knowledge included in their tools was very timely and focused more on the introductory phase, meaning that this tool was typically used shortly after the child was diagnosed with heart failure. However, participants did express that this tool could be provided to parents at times when they needed a review of the information. A participant explicitly stated, “the tool is mostly intended to be given at diagnosis but can be distributed for a refresher if needed” [I4].

Another participant highlighted that they also revise or add content to their tool based on trends from social media posts or parent inquiries to their foundation, highlighting that their tool was timely by addressing current parent questions, “Content in the tool is based on social media posts” [I1].

To also ensure that parents were not overloaded with too much information at the time of diagnosis, other key informants strategically placed knowledge in small chunks to avoid overwhelming parents. This was highlighted with the quote: “From our parent meetings, parents prefer knowledge in bite-size pieces” [I4]. This was a strategy that allowed parents to build on their knowledge rather than try to learn it all at once, demonstrating that key informants were aware of the huge learning curve that happens with parents who are in this situation.

#### Credible and Trustworthy Knowledge

All the participants in the interviews described the information presented in their tools as evidence-based. A participant shared that “guidelines are biggest go-to for information” [I2], meaning that they drew most of their information from published peer-reviewed material, along with some anecdotal knowledge from years of clinical experience. A second participant also confirmed that their tool was also “most based off medical guidelines” [I2 and I4].

Participants indicated that their information was mostly distributed in the hospital setting despite being posted on the web. They indicated that their information could be handed out in the form of printouts or families can be shown how to access digital or multimedia tools that were posted on their hospital webpage or reputable organization by hospital staff on the parent’s mobile device. A participant stated:

I share the website with the parent using their phone. They search on their phone, and I confirm it is the correct website. This is so they can find the information in the future.

I4

#### Challenges and Evolution in Knowledge

Despite the good intentions of health care professionals to share complex knowledge with parents in easier-to-understand formats, this piece is complex and presents challenges. Some of the challenges were issues related to the web-based sharing of information. A challenge that inherently comes with web-based knowledge sharing is optimizing search engines. A participant shared that their organization is working with the Google search engine as they acknowledged that their tool is not easily found on the first few pages of results, affecting the reach to their intended parent audience. They highlighted that they are “working with Google to improve their search optimization so parents can find their tools” [I4].

Another challenge faced by developers of the tools in our interviews is that the tools often do not include credible references, making it difficult for parents to discern whether the information is evidence-based. A participant acknowledged this, saying that “we do not include the references in our tools we distribute to families” [I3].

An additional challenge outlined in the interviews was related to having the tools available in only the English language when there are families where English is not their first language. This posed a challenge to the health care providers in the interviews because they felt that perhaps their tool was not as effective at translating that critical knowledge. At times, a participant stated that they would have to spend more time with the parents to ensure they understood the material because they could not read or write in the English language. A participant expressed, “Our Center has a large population of individuals fluent in Spanish. There are times we have read the pamphlets to families because they could not read English themselves” [I4].

The last challenge that participants outlined was making the time for refinements or updates to their tools. A participant described, “Heart failure is a complex disease so we are always looking to refine our tools.” [I2]. All participants acknowledged that they do not have regular set time intervals for editing and updating their tools. They all typically completed this task when they “thought about it” or when clinical practice changes occurred (I1, I2, I3, and I4). Some of the participants work with others who could alert them when updates were needed (eg, nursing staff or family comments) or simply relied on memory to update the documents.

## Discussion

### Principal Findings

This is the first ES to conduct a search for internet sources for parent audiences relating to children’s heart failure within North America. First, our ES identified that no apps exist on this topic, highlighting a significant knowledge gap for parents who are trying to seek information from this digital platform. Furthermore, our research highlighted that 17 web-based tools about children’s heart failure exist tools and were assessed to be adequate using SAM percent ratings.

We have highlighted that a modest number of relevant educational tools exist from our internet search (n=17), with varying degrees of content and health literacy for parent audiences. Notably, 88% (15/17) of the tools found appeared to be developed by clinicians for parents, instead of having parents actively involved in the development process. There were 2 tools that involved parent recommendations and feedback from a parent advisory group but were not created using parent experience evidence. Given the complexity of health journeys for families who have a child with heart failure, there is a critical need and gap to develop a tool based on parents’ lived experience to help deliver tools that are relevant and applicable to parent needs.

Of the relevant internet-based parent tools, no tool scored in the superior range, highlighting that work in the area of health communication and literacy could be improved upon. Most tools scored lower in the summary and review subsection, literacy subsection, and overall graphics section. Improving on these key aspects will provide parents, especially parents with lower literacy skills, information that is easier to understand with improved repetition of key information. However, the field of pediatric cardiology poses its own unique challenge in that this field contains many words with >2 to 3 syllables (eg, echocardiogram or cardiomyopathy). This aspect undoubtedly played a factor that increased the reading level and reduced the score in many of the tools. One of the key informants in the qualitative interviews highlighted their process for dealing with complex medical language to ensure that language was consistent, well defined in simpler terms, and providing information in *bite-size pieces* for parents and caregivers to enhance their uptake.

In the category of graphics, SAM scores identified that major refinements are needed in this area. An explanation for this lower score was that all relevant tools were website based and it was difficult to score these tools in relation to a *graphic cover* as suggested in the tools’ instructions. The recommended illustrations that Doak et al [16] outlined in their tool stated that simple line drawings can promote *realism* without distracting the details. As noted, this tool was developed in 1996 and intended mostly for print materials, so reviewers acknowledge that technology has advanced to include more digitalized, web-based infographics that can be just as impactful as line drawings. Infographics in today’s educational materials involve simpler computer infographics with more vivid, crisp colors that attracts the reader’s attention. Perhaps, updating of the assessment tool to include those aspects would have scored a few of the tools in the superior range, as reviewers found some of the graphics to be well done.

It is imperative that we improve knowledge translation strategies to improve the health literacy of parents and caregivers who care for children with heart failure in the home environment. Knowledge translation strategies that include parents as cocreators bring their unique perspective or lived experience that will improve uptake and understanding, as families in similar contexts will likely share similar knowledge needs [26]. When parents have a lack of understanding toward treatments or health conditions, worse outcomes occur in children’s health [27]. One way to mitigate these poor outcomes is to have robust health information available on the internet, and in alternative formats, that is based on research knowledge and parental lived experience. This is done through the avenues of improving parental and caregiver knowledge bases to make better decisions, reducing parental stress levels and invoking improved conversations with their child’s health provider through questions [28,29]. In addition, when clinicians are armed with credible and effective sources of information that can be easily shared with their parent audience, better relationships will result as parents will have more confidence in their health care provider.

As all tools did not include evidence-based references to indicate that they were developed from peer-reviewed research, the average parent would have difficulty discerning if the material from any of the tools were credible or even evidence-based. Recent published literature has demonstrated that a large portion of parents who searched the internet had difficulty discerning if the literature they found was from a credible source; furthermore, they were not confident in bringing it to a trusted health care professional [29]. Even more troubling is the fact that parents will make health decisions based on the information they find on the internet [28], which may or may not be based on the most credible sources. Despite clinicians’ good intentions of simplifying information to parents by not citing the source of their information, diligently citing evidence where they derived the material may relieve the stress of parents trying to discern whether the tool is evidence-based material.

### Limitations

As this search was conducted in July 2020, it is possible that more tools are now available to parents and caregivers or that the current tools scored in our search have now been updated to reflect different or enhanced content. As we know, the internet and app stores are rapidly adding more content or updating existing materials daily. Our search was only a snapshot in time and would be difficult to replicate the same results.

A limitation of our search was the use of only a single search engine (Google) to provide results, which may have exposed our results to an element of search bias. Published literature on search engines, such as Google, has suggested that theories relating to filter bubbles or personalized algorithms can change results based on who searches for health information [30-32]. Although we took steps to reduce this bias, by deleting cookies and turning off personalization, this is not a perfect process. Another solution would be to use engines, in addition to Google, in the web-based search process to provide more robust results.

Although the scoring of health information was performed using a validated SAM instrument, limitations still remain. We noticed that updates to the tool may be required in some sections that scored lower (eg, type of illustration) as the tool gave a higher score for simple adult-appropriate line drawings that are not congruent with today’s color infographics that can be generated from graphic artists. Current graphics are now designed as colorful infographics and characters, rather than simple line drawings or sketches, which the authors thought to be distracting. Scoring methods indicate that line drawings provide the least amount of distraction [16]. There would have been an improvement in scores if the tool had been updated to include simple computer infographics that are now commonly designed in current educational materials.

In addition, the 2 reviewers found the culture section in the instrument very difficult to score. This was evidenced by the similarity and lack of variability of scores. Culture within the context of children’s heart failure was very difficult to define within the context of pediatric heart failure solely based on a web-based tool or handout. Perhaps more detailed instructions and a definition for culture could be provided, making it more user-friendly. We did find that most graphics included varying types of races and genders among the photos of parents and children. Perhaps if there were tools included in video format, culture scores would vary more as there would be an increased presence of tone and gender role presentations.

### Conclusions

This ES sought to explore what multimedia educational information or tools existed on the internet and within app stores for parent audiences about children’s heart failure. From our search, we found 17 parent tools and no apps relating to children’s heart failure that were developed in Canada and the United States. This highlights a gap in knowledge for parents who prefer this type of web-based content for learning about this important topic. Using SAM scoring, most web-based tools scored overall in the adequate range, meaning that they were adequate to teach parents, but there are some key improvements, especially in reading level and graphics, that can be made to maximize their educational effectiveness. The qualitative interviews with key informants who developed the tools highlight three key themes: timely introductory knowledge, credible and trustworthy knowledge, and challenges and points in how organizations plan to evolve this knowledge in the future. Further research is required to evaluate the effectiveness of such parent-targeted tools and their impact on parents’ ability to learn and care for these children more confidently in the home setting.

## Appendix

Multimedia Appendix 1 Screening of apps and web-based tools.

Multimedia Appendix 2 List of included web-based pediatric heart failure tools (n=17).

Multimedia Appendix 3 Average overall Suitability Assessment of Materials percent rating for individual web-based tools.

Multimedia Appendix 4 Combined average Suitability Assessment of Materials percent rating score for all web-based tools (n=17) categorized by domain.

## Abbreviations

ES: environmental scan
SAM: Suitability Assessment of Materials
WCHRI: Women’s and Children’s Health Research Institute
