# Correction: Multimedia Knowledge Translation Tools for Parents About Childhood Heart Failure: Environmental Scan

**DOI:** 10.2196/39049

**Published:** 2022-06-21

**Authors:** Chentel Cunningham, Hyelin Sung, James Benoit, Jennifer Conway, Shannon D Scott

**Affiliations:** 1 Faculty of Nursing University of Alberta Edmonton, AB Canada; 2 Department of Pediatrics Faculty of Medicine and Dentistry University of Alberta Edmonton, AB Canada

In “Multimedia Knowledge Translation Tools for Parents About Childhood Heart Failure: Environmental Scan” (JMIR Pediatr Parent 2022;5(1):e34166) the authors noted one error.

In the originally published paper, Multimedia Appendix 1 contained one incorrect value. In the “Duplicate apps removed before screening” box, the value was listed as “6”.

In the corrected version of the paper, Multimedia Appendix 1 has been revised as follows:

In the “Duplicate apps removed before screening” box, the value is listed as “5”. The updated version of Multimedia Appendix 1 is available below.

The correction will appear in the online version of the paper on the JMIR Publications website on June 21, 2022, together with the publication of this correction notice. Because this was made after submission to PubMed, PubMed Central, and other full-text repositories, the corrected article has also been resubmitted to those repositories.

## Appendix

Multimedia Appendix 1 Corrected version of Multimedia Appendix 1: Screening of apps and web-based tools.

