# Supplementary material for: Multimedia Knowledge Translation Tools for Parents About Childhood Heart Failure: Environmental Scan
Source: JMIR Pediatr Parent. 2022 Mar 21;5(1):e34166. doi: 10.2196/34166 (PMC8981009; doi:10.2196/34166)
Supplement: Multimedia Appendix 1 [file pediatrics_v5i1e34166_app1.docx]

Multimedia Appendix 1: Screening of Application (Apps) & Web-based Tools.

**112** Apps identified from:

- Apple App Store (n = 50)
- Google Play Store (n = 53)
- Appeared in both stores (n=9)

**575** Internet tools identified from advanced Google^TM^ search:

- United States (n=300)
- Canada (n=275)

**Identification**

Duplicate apps removed before screening (n=5)

Duplicate internet tools removed before screening (n=120)

Internet tools screened for eligibility (n=455)

Apps screened for eligibility (n=107)

**Screening & Eligibility**

Internet Tools excluded (n=438)

- Content not related to Children’s Heart Failure (n=336)
- Target Audience (n=71)
- Not an educational tool (n=27)
- Development Origin (n=4)

Apps excluded (n=107)

- Content not related to pediatric heart failure (n=71)
- Not English Language (n=2)
- Development Origin (n=34)

Internet Tools included (n=17)

- Tools from Search (n=16)
- Tools identified from Subject Expert (n=1)

Apps included (n = 0)

**Included**
