# Supplementary material for: Multimedia Knowledge Translation Tools for Parents About Childhood Heart Failure: Environmental Scan
Source: JMIR Pediatr Parent. 2022 Mar 21;5(1):e34166. doi: 10.2196/34166 (PMC8981009; doi:10.2196/34166)
Supplement: Multimedia Appendix 2 [file pediatrics_v5i1e34166_app2.docx]

| Multimedia Appendix 2. List of Included Web-based Pediatric Heart Failure Tools (n=17). | | | | | | | |
| --- | --- | --- | --- | --- | --- | --- | --- |
| **Title** | **Author** | **URL** | **Location** | **Year of Last Update** | **Evidence Based** | **Format** |  |
| Congestive Heart Failure | About Kids Health | https://www.aboutkidshealth.ca/ | Canada | 2018 | No | Website |  |
| Congestive Heart Failure | Western Canadian Children’s Heart Network | http://www.westernchildrensheartnetwork.ca/ | Canada | 2014 | No | Handout/Brochure |  |
| Heart Failure in Children: Overview | A.D.A.M Health | https://www.healthing.ca/ | Canada | 2019 | Yes | Website |  |
| Heart Failure Fact Sheet | Barth Syndrome Foundation | http://www.barthsyndrome.ca/ | United States | 2015 | No | Handout/Brochure |  |
| Heart Failure in Children | Stanford Children’s Hospital | https://www.stanfordchildrens.org/ | United States | 2021 | No | Webpage |  |
| Congestive Heart Failure in Infants & Children | Cincinnati Children’s Hospital | https://www.cincinnatichildrens.org/ | United States | 2019 | No | Webpage |  |
| Heart Failure in Children | Children’s Hospital of Philadelphia | https://www.chop.edu/ | United States | 2021 | No | Webpage |  |
| Heart Failure in Children – Health Encyclopedia | University of Rochester | https://www.urmc.rochester.edu/ | United States | 2021 | No | Webpage |  |
| Pediatric Heart Failure | Uchicago: Comer Children’s | https://www.uchicagomedicine.org/ | United States | Not listed | No | Webpage |  |
| When Your Children Has Congestive Heart Failure (CHF) | Fairview | https://www.fairview.org/ | United States | 2020 | No | Webpage |  |
| Heart Failure in Children | Medline Plus/US National Institute of Medicine | https://medlineplus.gov/ | United States | 2021 | Yes | Webpage |  |
| Preventing Heart Failure in Children | Seconds Count | http://www.secondscount.org/ | United States | 2015 | No | Webpage |  |
| Congestive Heart Failure | Children’s Hospital of Chicago | https://www.luriechildrens.org/ | United States | 2021 | No | Webpage |  |
| Pediatric Heart Failure: A Guide for Parents and Families | Children’s Cardiomyopathy Foundation | https://www.childrenscardiomyopathy.org/ | United States | 2020 | No | Handout/Brochure |  |
| Heart Failure in Children - Overview | University of Florida Health | https://ufhealth.org/ | United States | 2018 | Yes | Webpage |  |
| Understanding Heart Failure in Children | Children’s Hospital of New Orleans/LCMC Health | https://www.chnola.org/ | United States | 2018 | No | Webpage |  |
| An Introduction to Pediatric Heart Failure | Action Learning Network | https://myactioneducation.org/ | United States | 2021 | No | Webpage/Handout |  |
