# Supplementary material for: Multimedia Knowledge Translation Tools for Parents About Childhood Heart Failure: Environmental Scan
Source: JMIR Pediatr Parent. 2022 Mar 21;5(1):e34166. doi: 10.2196/34166 (PMC8981009; doi:10.2196/34166)
Supplement: Multimedia Appendix 3 [file pediatrics_v5i1e34166_app3.docx]

| Multimedia Appendix 3. Average Overall SAM Percent Rating for Individual Web-based Tools. | |
| --- | --- |
| **Web ID** | **Average Rating, n (%)** |
| Web001 | 25.5 (61.7) |
| Web002 | 20 (47.6) |
| Web003 | 21.5 (51.2) |
| Web004 | 16 (38.1) |
| Web005 | 24.5 (58.3) |
| Web006 | 17.5 (41.7) |
| Web007 | 16 (38.1) |
| Web008 | 19.5 (46.4) |
| Web009 | 18.5 (44.0) |
| Web010 | 19.5 (46.4) |
| Web011 | 19.5 (46.4) |
| Web012 | 17.5 (41.7) |
| Web013 | 16.5 (39.3) |
| Web014 | 23.5 (56.0) |
| Web015 | 22 (52.4) |
| Web016 | 24.5 (58.3) |
| Web017 | 26 (62.0) |
