# Supplementary material for: Multimedia Knowledge Translation Tools for Parents About Childhood Heart Failure: Environmental Scan
Source: JMIR Pediatr Parent. 2022 Mar 21;5(1):e34166. doi: 10.2196/34166 (PMC8981009; doi:10.2196/34166)
Supplement: Multimedia Appendix 4 [file pediatrics_v5i1e34166_app4.docx]

| Multimedia Appendix 4. Combined Average SAM Percent Rating Score for All Web-based Tools (n=17) Categorized by Domain. | | | |
| --- | --- | --- | --- |
| SAM Factor | Score, n (%) | | |
|  | Superior | Adequate | Not Suitable |
| **Content** | | | |
| Purpose | 8 (47.1) | 4.5 (26.5) | 4.5 (26.5) |
| Content Topics | 2 (11.8) | 11.5 (67.5) | 7 (41.2) |
| Summary & Review | 1.5 (8.8) | 1 (5.9%) | 14 (82.4) |
| **Literacy Demand** | | | |
| Reading Grade Level | 0 (0.0) | 4 (23.5.) | 13 (76.5) |
| Writing Style | 7 (41.2) | 11 (64.7) | 0.5 (2.9) |
| Sentence Construction | 12.5 (73.5) | 4.5 (26.5) | 0.5 (5.9) |
| Vocabulary | 5 (29.4) | 6.5 (61.1) | 1 (5.9) |
| Learning Aids | 16 (94.1) | 0 (0.0) | 1 (5.9) |
| **Graphics** | | | |
| Cover Graphics | 0.5 (2.9) | 4.5 (26.5) | 12.5 (73.5) |
| Type of Illustrations | 0.5 (2.9) | 5 (29.4) | 10.5 (61.2) |
| Relevance of Graphics | 2.5 (14.7) | 5.5 (32.4) | 10.5 (61.2) |
| Graphic Explanation | 0 (0.0) | 0 (0.0) | 17 (100) |
| Graphic Caption | 1 (5.9) | 0 (0.0) | 16 (94.1) |
| **Layout & Type** | | | |
| Typography | 17 (100.0) | 0 (0.0) | 0 (0.0) |
| Layout | 10.5 (61.2) | 7 (41.2) | 1 (5.9) |
| Subheadings | 7 (41.2) | 3.5 (20.6) | 6.5 (38.2) |
| **Learning Simulation & Motivation** | | | |
| Interactions Included | 1 (5.9) | 6.5 (38.2) | 9.5 (55.9) |
| Behaviour Changes Modelled | 2 (11.8) | 9.5 (55.9) | 6 (35.3) |
| Motivation | 11.5 (67.6) | 5.5 (32.3) | 0 (0.0) |
| **Cultural Appropriateness** | | | |
| Logic, Language, Experience (LLE) | 17 (100.0) | 0 (0.0) | 0 (0.0) |
| Cultural Image & Examples | 0 (0.0) | 17 (100) | 0 (0.0) |
